# Incidence of serious infections in patients with ANCA-associated vasculitis receiving immunosuppressive therapy: A systematic review and meta-analysis

**DOI:** 10.3389/fmed.2023.1110548

**Published:** 2023-03-01

**Authors:** Athanasios Vassilopoulos, Stephanos Vassilopoulos, Markos Kalligeros, Fadi Shehadeh, Eleftherios Mylonakis

**Affiliations:** ^1^Infectious Diseases Division, Rhode Island Hospital, Providence, RI, United States; ^2^Warren Alpert Medical School of Brown University, Providence, RI, United States; ^3^School of Electrical and Computer Engineering, National Technical University of Athens, Athens, Greece

**Keywords:** ANCA-associated vasculitis, azathioprine, cyclophosphamide, maintenance, methotrexate, mycophenolate mofetil, serious infections, rituximab

## Abstract

**Introduction:**

Rituximab and azathioprine are used to induce or maintain remission in patients with ANCA-associated vasculitis (AAV). We evaluated the incidence of serious infections and infection-related deaths in patients with AAV treated with rituximab and azathioprine, during the maintenance of remission period.

**Methods:**

We searched PubMed and EMBASE for randomized clinical trials (RCTs) and observational studies evaluating immunosuppressive agents in patients with AAV. We defined serious or severe infections according to the National Cancer Institute Common Terminology Criteria for Adverse Events (CTCAE) version 5.0. The study was registered on PROSPERO (CRD42022366269).

**Results:**

From 1,265 abstracts, we identified 21 studies (7 RCTs and 14 observational), with relevant data. We included data from 1,284 and 2,938 individuals for assessment in our primary and secondary outcomes, respectively. The overall cumulative incidence of serious infections was 15.99% (CI 95%: 6.95–27.53%) during the total follow-up period (induction and maintenance) and 7.62% (CI 95%: 4.43–11.43%) during the maintenance period. Additionally, we found a 0.49% overall case fatality rate (CI 95%: 0.02–1.37%) and a 0.09% infection-related mortality rate (CI 95%: 0.00–0.51%) during maintenance treatment. Notably, we found a 14.61% (CI 95%: 10.19–19.61%) cumulative incidence of serious infections among patients who received rituximab and a 5.93% (CI 95%: 1.19–13.26%) cumulative incidence of serious infections among patients who received azathioprine during maintenance. Moreover, the cumulative incidence of serious infections during the total follow-up period (induction and maintenance) was 20.81% (CI 95%:4.56–43.70%) for the combination of cyclophosphamide and azathioprine and 14.12% (CI 95%: 5.20–26.00%) for rituximab.

**Discussion:**

The cumulative incidence of serious infections during total follow-up and maintenance was within expected limits, while fatal infections during maintenance treatment were uncommon. Additionally, treatment with rituximab for both induction and maintenance did not exceed the anticipated by previous studies incidence of serious infections. Clinical practice and long-term follow up data are needed to corroborate these findings.

**Systematic review registration:**

Identifier: PROSPERO (CRD42022366269).

## 1. Introduction

Anti-neutrophil cytoplasmic antibody (ANCA)-associated vasculitides (AAV) affect small- and medium-sized blood vessels (1). This multi-system autoimmune disease includes three categories depending on clinical presentation: granulomatosis with polyangiitis (GPA), microscopic polyangiitis (MPA), and eosinophilic GPA (EGPA). Disease manifestation is frequently associated with loss of tolerance to proteinase 3 (PR3) or myeloperoxidase (MPO), or other neutrophil primary granule proteins (1). Additionally, clinical features in PR3-AAV and MPO-AAV may differ (1).

AAV are associated with life-threatening complications usually due to disease-related organ damage or adverse events of treatment regimens (2, 3). The goal of therapy for AAV is to achieve remission within 3–6 months and prevent relapse with the least toxicity possible (4). The American College of Rheumatology's treatment guidelines for patients with GPA or MPA, have recently shifted toward newer agents for both induction and maintenance (4). More specifically, even though, induction regimens with cyclophosphamide and glucocorticoids have been the mainstay of treatment (5), rituximab, has shown similar efficacy in newly diagnosed patients and superior efficacy in relapsing disease when compared to cyclophosphamide (6). Following induction, maintenance of remission in patients with severe disease is usually achieved by the administration of azathioprine, or rituximab (4). Other agents such as methotrexate (7) and mycophenolate mofetil (8) are usually reserved for patients with non-severe limited disease (4).

Infectious complications are some of the most common adverse outcomes in patients with AAV (9). Despite the use of appropriate preventive measures including vaccination, co-administration of prophylaxis for Pneumocystis pneumonia, and decreasing the dose of glucocorticoids, serious infections continue to be a prevalent and substantial concern (2, 3, 9). Importantly, the disease process itself, which frequently involves the upper and lower respiratory tract, contributes to the development of serious infection (3). In addition, available treatment regimens for AAV (including use of high dose glucocorticoids in the induction period) increase infection susceptibility *via* various mechanisms (10–13).

A recent meta-analysis evaluated the incidence of serious infections in rituximab-treated patients mainly during the induction period and reported a rate of 15.40% (14). However, there is limited information regarding the risk for serious infection during the maintenance period compared to the induction period. We performed a systematic review and meta-analysis of randomized clinical trials (RCTs) and observational studies to calculate the incidence of serious infections and deaths attributed to infections in patients with AAV across different treatment regimens during the maintenance as well as the total (induction and maintenance) follow-up period.

## 2. Methods

### 2.1. Data sources and search strategy

For this systematic review and meta-analysis, we adhered to the Preferred Reporting Items for Systematic Reviews and Meta-Analyses (PRISMA) statement checklist (15) and registered our study on PROSPERO (CRD42022366269). As per protocol, we searched PubMed/MEDLINE and EMBASE databases for RCTs and observational studies published in English with last access on September 27, 2022. We utilized the following search term: (ANCA OR ANCA-associated OR “anti-neutrophil cytoplasmic antibody” OR AAV) AND (rituximab OR cyclophosphamide OR azathioprine OR methotrexate OR “mycophenolate mofetil” OR MTX OR AZA OR CYC OR MMF OR RTX) AND (randomized OR observational OR prospective OR retrospective).

### 2.2. Study selection

We considered an RCT or observational study to be eligible for any of our outcomes of interest if it included adult patients with AAV treated with a combination of treatment regimens for induction and maintenance of remission, and it included extractable data on any outcome of interest. To achieve a more homogeneous cohort, we did not include data from patients with EGPA in our analysis due to their different natural history and treatment (1, 4). Therefore, we included patients with GPA, MPA, and renal-limited disease. The agents we evaluated were cyclophosphamide, rituximab, azathioprine, mycophenolate mofetil, and methotrexate.

### 2.3. Definitions

The definition of serious or severe infections included Grade 3–5 infectious complications based on the National Cancer Institute Common Terminology Criteria for Adverse Events (CTCAE) version 5.0 (16). More specifically, a Grade 3 infectious adverse event is defined as the need for intravenous antibiotics or invasive intervention, Grade 4 refers to life-threatening consequences that necessitate immediate intervention, and Grade 5 refers to death due to an infectious cause (16).

### 2.4. Outcomes

We included eligible studies that used a treatment regimen that was present in an adequate number of studies (>2). The cumulative incidence of serious infections during the maintenance period overall and stratified by the prescribed agent's mechanism of action was the primary outcome of our analysis. Secondary outcomes were: (a) case fatality rate attributable to infection during the maintenance period stratified by treatment regimen prescribed, (b) patient infection-related mortality rate during maintenance period stratified by the administered agent, and (c) cumulative incidence of serious infections during the induction and maintenance period (total follow-up) among different treatment regimens. Of note, we also analyzed outcomes separately for RCTs to provide a higher level of evidence because they have the lowest chance of bias (17).

The proportion of patients with AAV and serious infection who died from the infection was defined as the case fatality rate. In terms of mortality rate, we defined it as the proportion of patients with AAV who died from infection in relation to the total available number of individuals with AAV during the maintenance period. Finally, we classified patients in the rituximab treatment subcategory if they received rituximab as a monotherapy or in combination with another immunosuppressive agent.

### 2.5. Data extraction and quality assessment

To assess eligibility, two reviewers (AV and SV) independently screened titles and abstracts. The two reviewers independently retrieved and evaluated the full text of selected articles and disagreements were resolved through discussion with another author, FS, and consensus. For each study, data on patient population, interventions, outcomes of interest, and quality were independently extracted. The extracted information also included the main characteristics of each study (author, publication year), proportion of population with GPA and MPA, proportion of population with PR-3 ANCA and MPO ANCA positivity at diagnosis, proportion of males, number of patients in each treatment arm, duration of total follow-up and maintenance. For our analysis, we also extracted the number of serious infections, their causes, and the number of patients affected over the course of the total follow-up as well as during the maintenance period alone. Also, the number of deaths attributable to infections over the course of the maintenance period was evaluated.

For methodological quality, the risk of bias of RCTs was assessed with the Revised Cochrane risk-of-bias tool (RoB 2). We evaluated the randomization process, deviations from the intended interventions, the extent of missing outcome data, outcome measurement, and outcome selection (18). Regarding the risk-of-bias of the observational studies, we used the risk of bias in non-randomized studies of interventions (ROBINS-I) tool. We assessed confounding, participant selection, intervention classification, deviations from intended interventions, missing data, outcome measurement, and the selection of the reported result (19). The synthesis of our risk-of-bias figures was performed using a visualization tool, the online software robvis (20).

To assess the quality of evidence, we utilized the Grading of Recommendations, Assessment, Development, and Evaluation (GRADE) framework (21). Based on GRADE, the quality of evidence and their stratification was graded as high, moderate, low, or very low. “Quality of evidence” was reduced based on 5 factors: study design limitations, inconsistency, indirectness, imprecision, and publication bias. After using the RoB2 and ROBINS-I tools to assess risk of bias, we would downgrade the quality of evidence by 1 or 2 levels in terms of study design limitations if moderate or high risk of bias was present, respectively, in at least one included study. Regarding “inconsistency,” quality of evidence in each of our outcomes would be downgraded by one level if I2 was higher than 70% or not available. If serious infection or an infection-related death was not diagnosed based on the National Cancer Institute Common Terminology Criteria for Adverse Events (CTCAE) we would downgrade “indirectness” by one level. Given the nature of our results, which are not dependent on the basis of the trade-off between desirable and undesirable outcomes, “imprecision” was a factor that could not be affected in our analyses of pooled proportions and their confidence intervals. Finally, “quality of evidence” was downgraded if publication bias was strongly suspected or unclear. The quality of evidence in our outcomes would be upgraded after evaluating effect size and dose effect if no downgrade was performed on the previous factors. However, both upgrading factors were not applicable in our analyses, because our pooled estimates are proportions.

### 2.6. Statistical analysis

For data analysis, the Stata v17 software (Stata Corporation, College Station, TX) was used. In order to estimate the cumulative incidence of serious infections among patients with AAVs during the maintenance period, we stratified patients with AAV by the maintenance agent they received and performed a random effects meta-analysis using the DerSimonian and Laird approach (22). In order to achieve admissible confidence intervals for each individual study as well as for the pooled proportion, we used the Freeman Tukey double arcsine transformation (23, 24).

Notably, a random effects model was selected (22) due to expected differences in the proportion of population with GPA/MPA, the proportion of PR3/MPO ANCA positivity at diagnosis and the median duration of total and maintenance follow-up. More specifically, we expected a difference in the proportion of patients with GPA and MPA, as well as PR3 and MPO positivity, because the geographical prevalence of these diseases varies (25), while PR3 and MPO positivity are positively related to GPA and MPA, respectively (26). Furthermore, we used random effects because treatment and follow-up duration could differ across included studies. Additionally, we conducted a meta-regression analysis to investigate the extent of the differences in study characteristics and their correlation with the heterogeneity between studies (27).

We used the I2 statistic to estimate heterogeneity between included studies in all outcomes, and the Egger's test to investigate publication bias and small study effects (28, 29). For the interpretation of heterogeneity with the I2 statistic we used the approach detailed as follows: I2 values of 25, 50, and 75% represent low, moderate, and high heterogeneity, respectively (28). We set statistical significance at α = 0.05.

## 3. Results

### 3.1. Search results and study characteristics

**Figure 1:**
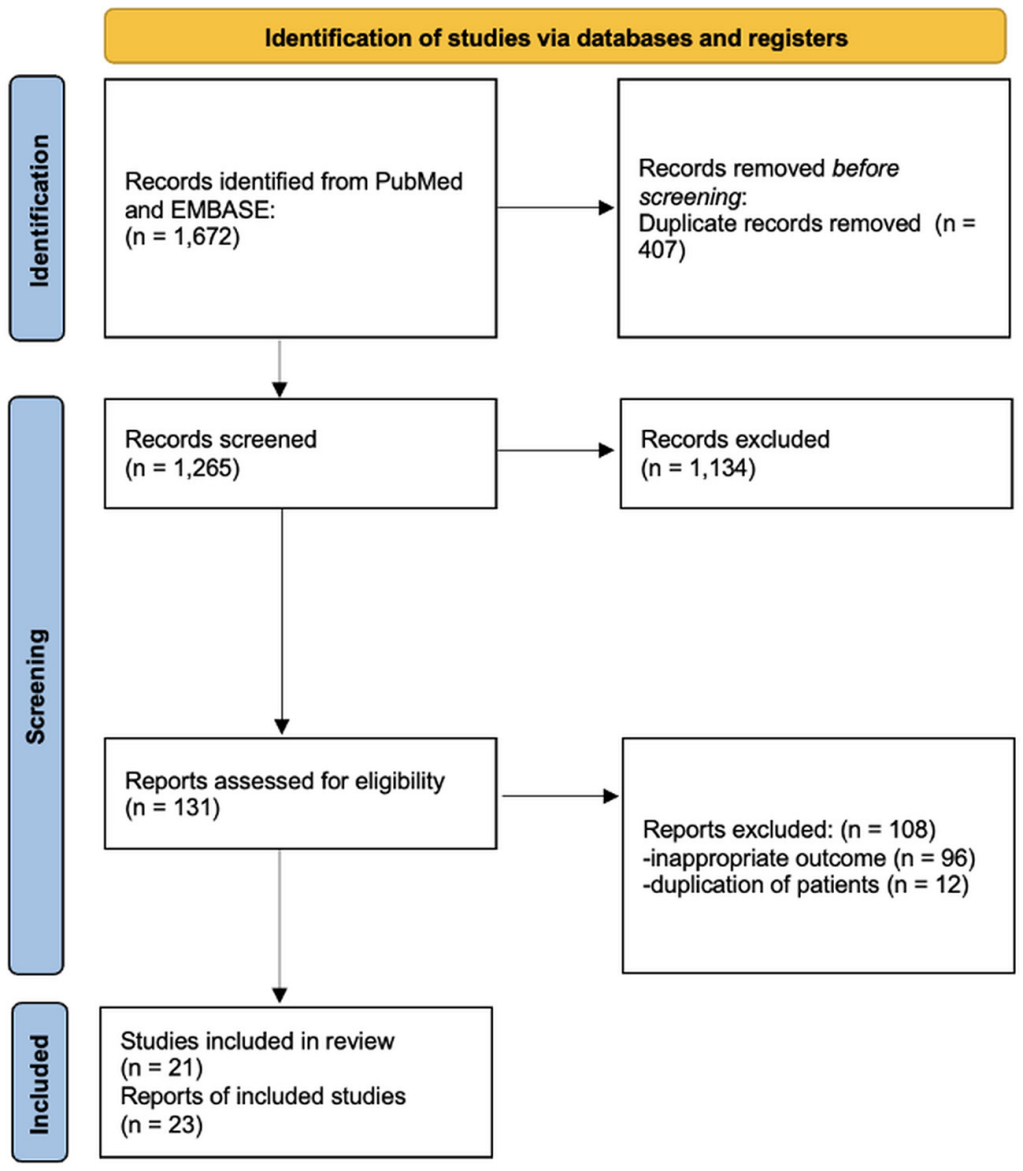
Flow diagram for selection of studies included in the systematic review and meta-analysis.

After deduplicating the results of the literature search in PubMed and EMBASE, we assessed 1,265 abstracts. After excluding a total of 1,134 studies based on their title and abstract, 131 publications were evaluated in full text. For the analysis of our primary outcome, 21 studies were retrieved (Figure 1). Specifically, we found 7 RCTs, and 14 observational studies (10 retrospective and 4 prospective) (6, 7, 30–48). Two RCTs reported extension follow-up data in additional publications (49, 50). Additionally, we found 13 studies that did not report data on our primary outcome but included data on secondary outcomes. In detail, we found 7 RCTs (1 RCT extension follow-up publication), as well as five retrospective and one prospective observational study (51–64).

In Table 1 the baseline characteristics of the studies that we included in our primary outcome are presented. We found 412 patients treated with azathioprine (12–42 months) (7, 30, 32, 45, 49, 50) and 617 with rituximab (12–64.8 months) (33, 36, 38, 40, 42–44, 46, 47). Regarding other agents, we identified 206 patients treated with methotrexate (12–25.2 months) (30, 34, 37, 41), and 49 with mycophenolate mofetil (12–33 months) (35, 39, 45, 48). Five RCTs (7, 32–48, 50) and 14 observational studies provided extractable data on the type of serious infection. We identified 9 RCTs and 23 studies in total (7, 30, 32–43, 45, 46, 48–51, 58, 62, 63) that reported infection-related causes of death. Overall, after accounting for secondary outcomes, we analyzed 2,938 patients (51–64).

**Table 1:** Study characteristics.

**Study**	**Study type**	**Follow-up duration (months)**	**Induction drug**	**Maintenance drug**	**Maintenance duration (months)**	**Patients (*N*)**	**Age at baseline (years)**	**Creatinine concentration**	**Renal disease (*N*)**	**Lung disease (*N*)**	**TMP/SMX (*N*)**
Terrier et al. (50) MAINRITSAN	RCT	60	CYC	RTX	14	57	54 ± 13 (mean, SD)	118.2 ± 73.1 μmol/l (mean, SD)	40	9	NA
Terrier et al. (50) MAINRITSAN	RCT	60	CYC	AZA	18	58	56 ± 14 (mean, SD)	129.6 ± 70.3 μmol/l (mean, SD)	41	11	NA
Charles et al. (33) MAINRITSAN2	RCT	28	CYC/RTX/MTX	RTX	18	81	62 ± 14 (mean, SD)	NA	60	50	81
Charles et al. (33) MAINRITSAN2	RCT	28	CYC/RTX	RTX	18	81	59 ± 13 (mean, SD)	NA	56	44	81
Hiemstra et al. (7) IMPROVE	RCT	48	CYC	AZA	42	80	55.1 ± 15.2 (mean, SD)	2.9 mg/dl (1.1–3.5) (median, IQR)	NA	NA	NA
Tuin et al. (32)	RCT	48	CYC	AZA	18	43	60 ± 11 (mean, SD)	1.1 mg/dl (0.9–1.8) (median, IQR)	32	24	43
Tuin et al. (32)	RCT	48	MMF	AZA	18	41	60 ± 13 (mean, SD)	1.3 mg/dl (1–1.7) (median, IQR)	31	18	41
Specks et al. (49)	RCT	18	CYC	AZA	15	98	51.5 ± 14.1 (mean, SD)	NA	66	53	98
RAVE											
Pagnoux et al. (30) WEGENT	RCT	NA	CYC	MTX	12	63	59.8 ± 11.9 (mean, SD)	1.4 ± 0.61 mg/dl (mean, SD)	48	41	NA
Pagnoux et al. (30) WEGENT	RCT	NA	CYC	AZA	12	63	56.3 ± 13.8 (mean, SD)	1.52 ± 1.15 mg/dl (mean, SD)	47	52	NA
Metzler et al. (34)	RCT	24	CYC	MTX	18	28	54 (25–67) (median, range)	NA	NA	NA	NA
Azar et al. (45)	Retrospective	23	RTX	MTX	23	11	NA	NA	NA	NA	NA
Azar et al. (45)	Retrospective	23	RTX	AZA	23	29	NA	NA	NA	NA	NA
Azar et al. (45)	Retrospective	23	RTX	MMF	23	7	NA	NA	NA	NA	NA
Besada et al. (44)	Retrospective	47	RTX	RTX	47	35	50 (14–79) (median, range)	NA	21	22	NA
Carranza-Enríquez et al. (43)	Retrospective	19	NA	RTX	19	24	49 (33–64) (median, IQR)	NA	NA	NA	NA
Charles et al. (42)	Retrospective	18	NA	RTX	18	50	NA	NA	NA	NA	NA
Gayatri et al. (40)	Prospective	24	RTX	RTX	24	21	51 (34–58) (median, IQR)	NA	3	10	NA
Kazderova et al. (39)	Retrospective	12	NA	MMF	12	15	NA	NA	15	NA	NA
Pendergraft III et al. (38)	Retrospective	25.2	NA	RTX	25.2	172	60 ± 16 (mean, SD)	1.3 mg/dl (1–2.1) (median, IQR)	106	75	NA
Roubaud-Baudron et al. (36)	Retrospective	38	NA	RTX	38	28	50.5 (19–78) (median, IQR)	NA	9	18	NA
Thomas et al. (47)	Retrospective	64.8	NA	RTX	64.8	43	NA	NA	NA	NA	NA
Ayan et al. (46)	Retrospective	24	RTX	RTX	13	25	44 (30–54) (median, IQR)	NA	14	16	13
de Groo et al. (41)	Retrospective	18	NA	MTX	18	33	NA	NA	NA	NA	0
Reinhold-Keller et al. (37)	Prospective	24	CYC	MTX	19	71	NA	NA	NA	NA	NA
Silva et al. (35)	Prospective	18	MMF	MMF	12	13	NA	NA	NA	NA	13
Langford et al. (48)	Prospective	18	CYC	MMF	12	14	49 (37–61) (median, IQR)	NA	6	8	14

AZA, azathioprine; CYC, cyclophosphamide; IQR, interquartile range; MMF, mycophenolate mofetil; MTX, methotrexate; *N*, number of patients; NA, not available; RCT, randomized clinical trial; RTX, rituximab; SD, standard deviation; TMP/SMX, trimethoprim-sulfamethoxazole.

### 3.2. Patients with serious infections during the maintenance period

**Figure 2:**
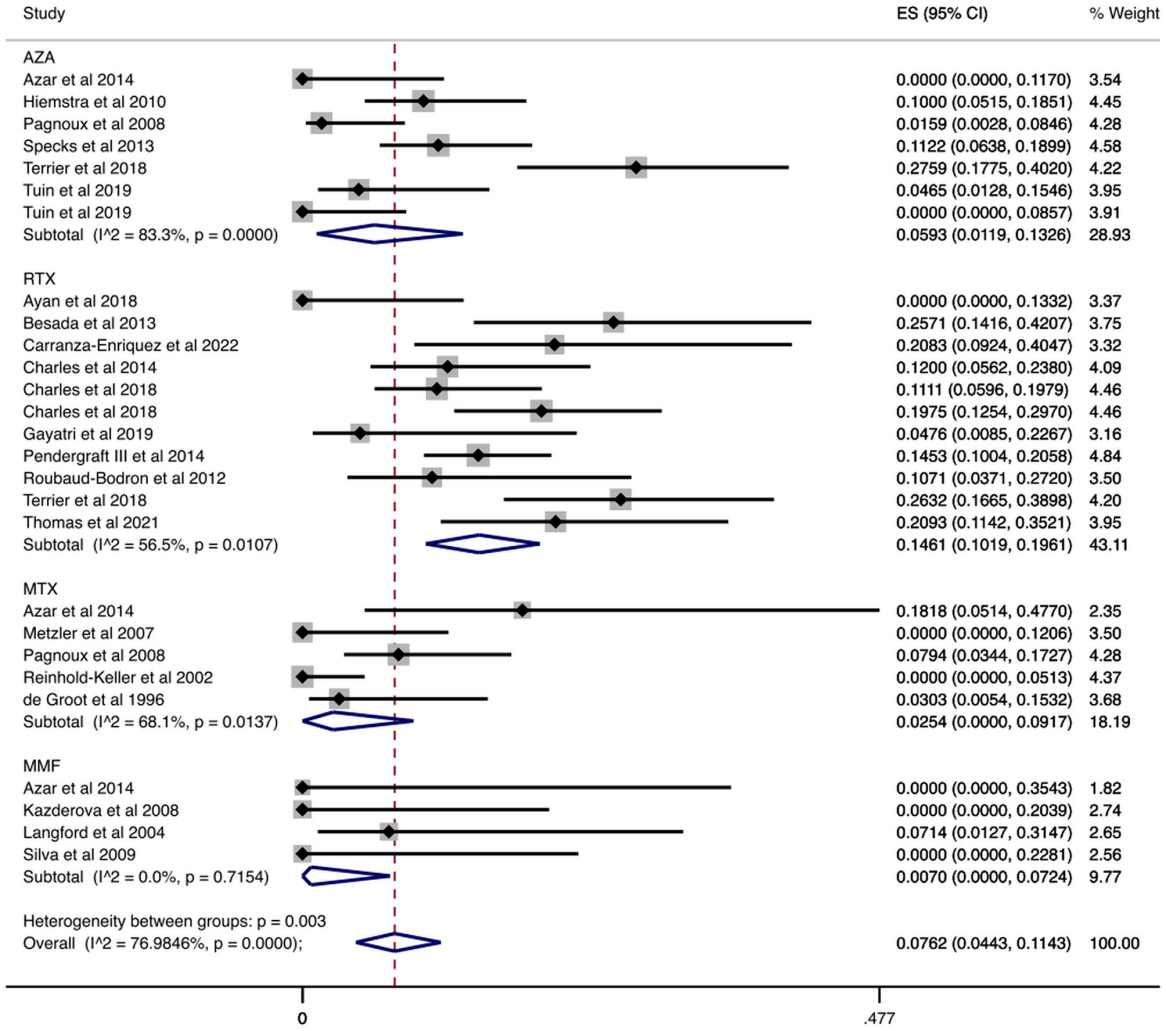
Serious infections cumulative incidence during the maintenance period. Serious infections overall and stratified by agent administered during the maintenance period. Individual and combined estimates of the cumulative incidence of serious infections with 95% confidence intervals. ES, Effect Size (Cumulative incidence).

We examined data from 1,284 patients in studies that reported the number of patients who had serious infections during the maintenance period. In Table 2 and Figure 2 the cumulative incidences of serious infections identified during the maintenance period are presented. The overall cumulative incidence of serious infections was 7.62% (CI 95%: 4.43–11.43%). The cumulative incidence of serious infections in patients treated with azathioprine was 5.93% (CI 95%: 1.19–13.26%), while it was 14.61% (CI 95%: 10.19–19.61%) in patients treated with rituximab. Regarding other agents used in limited disease, the cumulative incidence of serious infections in patients treated with methotrexate was 2.54% (CI 95%: 0.00–9.17%) and 0.70% (CI 95%: 0.00–7.24%) in mycophenolate mofetil-treated patients. Also, we performed a meta-regression analysis in each administered agent and overall, for the proportion of patients with GPA and MPA, and total number of patients in each study. No correlation was found between the cumulative incidence of serious infections and these variables (Supplementary Table 1).

**Table 2:** (A) Cumulative incidence of serious infections during the maintenance period, (B) Cumulative incidence of serious infections during total follow-up, (C) Case fatality rate attributable to infection during the maintenance period, (D) Infection-related mortality rate during the maintenance period.

**Treatment period**	**Patient number**		**CI 95%**
**A. Maintenance**		**Cumulative incidence**	
Rituximab	617	14.61%	10.19–19.61%
Rituximab RCTs	219	18.32%	10.46–27.70%
Azathioprine	412	5.93%	1.19–13.26%
Azathioprine RCTs	383	7.33%	1.82–15.58%
Methotrexate	206	2.54%	0.00–9.17%
Methotrexate RCTs	91	4.30%	0.72–9.91%
Mycophenolate mofetil	49	0.70%	0.00–7.24%
**Overall RCTs**	693	9.19%	4.50–15.16%
**Overall**	1,284	7.62%	4.43–11.43%
**B. Induction** **+** **Maintenance**		**Cumulative incidence**	
Cyclophosphamide + Azathioprine	522	20.81%	4.56–43.70%
Cyclophosphamide + Azathioprine RCTs	124	12.50%	5.64–21.19%
Mycophenolate mofetil + Azathioprine RCTs	111	14.99%	8.78–22.41%
Rituximab + Rituximab	181	14.12%	5.20–26.00%
Overall RCTs	235	12.59%	4.66–23.16%
Overall	814	15.99%	6.95–27.53%
**C. Maintenance**		**Case fatality rate**	
Rituximab	514	1.29%	0.03–3.67%
Rituximab RCTs	219	0.22%	0.00–1.70%
Azathioprine RCTs	342	0.30%	0.00–1.50%
Methotrexate	107	0.22%	0.00–3.15%
Overall RCTs	561	0.27%	0.00–1.12%
Overall	963	0.49%	0.02–1.37%
**D. Maintenance**		**Mortality rate**	
Rituximab	665	1.01%	0.05–2.70%
Rituximab RCTs	219	0.22%	0.00–1.70%
Azathioprine	540	0.01%	0.00–0.59%
Azathioprine RCTs	511	0.01%	0.00–0.63%
Methotrexate	206	0.01%	0.00–1.35%
Methotrexate RCTs	91	0.75%	0.00–4.37%
Mycophenolate mofetil	49	0.00%	0.00–4.14%
Overall RCTs	821	0.07%	0.00–0.62%
Overall	1,460	0.09%	0.00–0.51%

CI, confidence intervals; RCT, randomized clinical trial.

**Figure 3:**
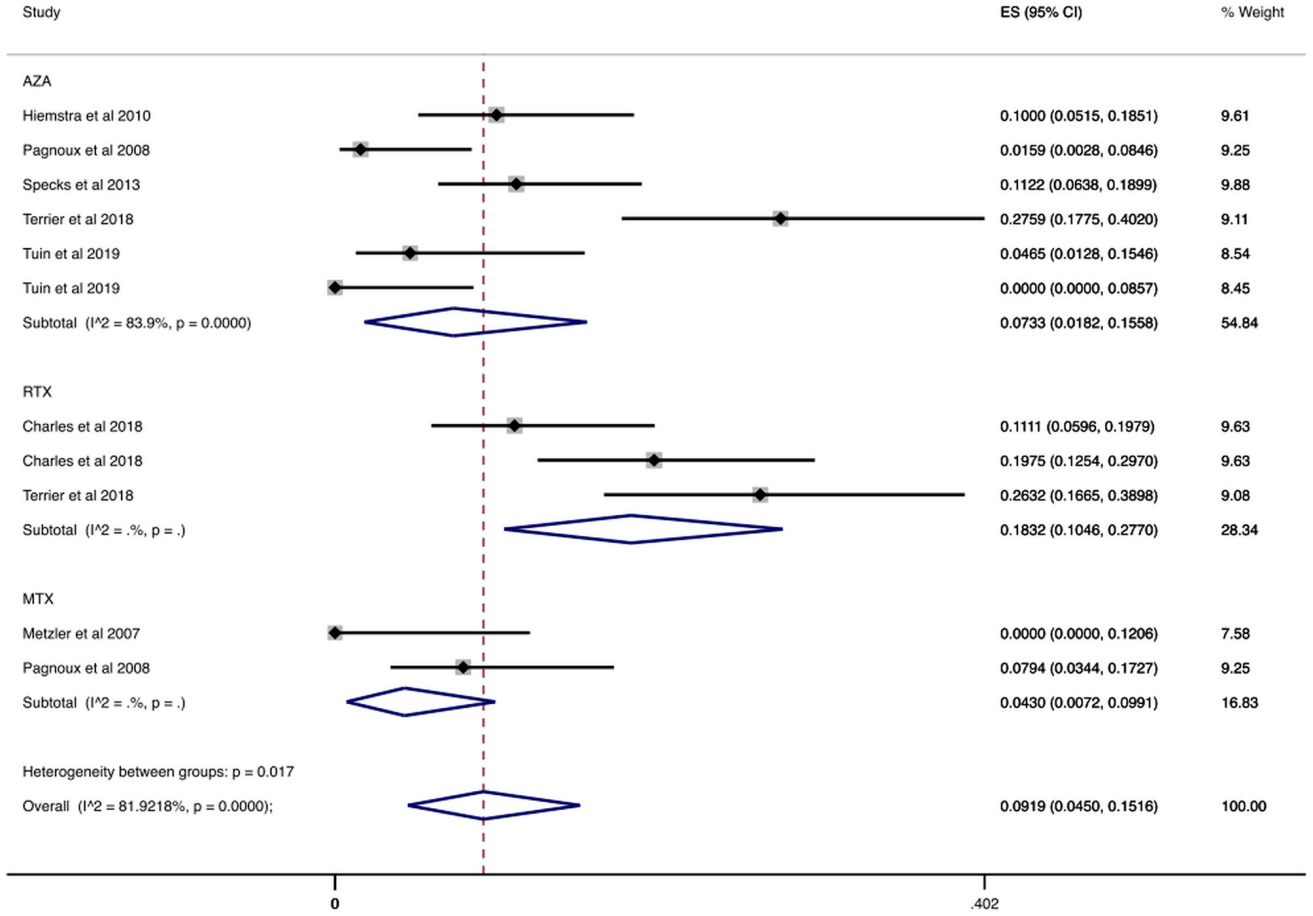
Serious infections cumulative incidence during maintenance period in RCTs. Serious infections overall and stratified by agent administered during the maintenance period in RCTs. Individual and combined estimates of the cumulative incidence of serious infections with 95% confidence intervals. ES, Effect Size (Cumulative incidence).

In a sub-analysis that focused on patients enrolled in RCTs, we analyzed 693 patients enrolled in RCTs that reported the number of patients who had serious infections during the maintenance period. In these studies, the maintenance period ranged from 12 to 42 months. Our results are summarized in Table 2 and Figure 3. Specifically, the overall cumulative incidence of serious infections among eligible patients was 9.19% (CI 95%: 4.50–15.16%). We found the incidence of serious infection to be 7.33% (CI 95%: 1.82–15.58%), and 18.32% (CI 95%: 10.46–27.70%) among patients receiving azathioprine and rituximab, respectively. As for agents used in non-severe disease, the cumulative incidence of serious infections in methotrexate-treated patients was 4.30% (CI 95%: 0.72–9.91%).

In Table 3 we present the reported causes of serious infection during the maintenance period in 14 observational studies and 5 RCTs (7, 32–48, 50). More than half of the serious infections (53%, 78/147) involved the respiratory tract and especially pneumonia that was diagnosed in 37 cases. In the 5 RCTs, the respiratory tract was also the site of nearly half of the serious infections (47%, 46/98), with 19 cases of pneumonia (7, 32–34, 50). Sepsis was relatively uncommon (6/147), but the mortality was high (discussed below).

**Table 3:** Serious infection causes during the maintenance period.

**Serious infection causes**	**All studies**	**RCTs**	**Death**	**All studies**	**RCTs**
Respiratory	78	46	Respiratory	7	1
Pneumonia	37	19	Sepsis	6	3
*Pneumocystis jirovecii* pneumonia	3	2	Unspecified	1	0
Bronchitis	21	21	Total	14	4
Other respiratory	20	6			
Gastrointestinal	16	13			
Genitourinary	7	6			
Sepsis	6	6			
Other	13	8			
Unspecified	27	9			
Total	147	98			

RCT, randomized clinical trial. Other in RCTs: Two cases of herpes zoster, and 1 case each of bacterial orchitis, bacterial elbow hygroma, bacterial endocarditis, ENT infection, paronychia, osteomyelitis. Other in all studies: 3 cases of herpes zoster, and 1 case each of bacterial orchitis, bacterial elbow hygroma, bacterial endocarditis, ENT infection, paronychia, osteomyelitis, erysipelas, cutaneous abscess, otitis media, mucositis.

### 3.3. Secondary outcomes

#### 3.3.1. Case fatality rate attributable to infection during the maintenance period

**Figure 4:**
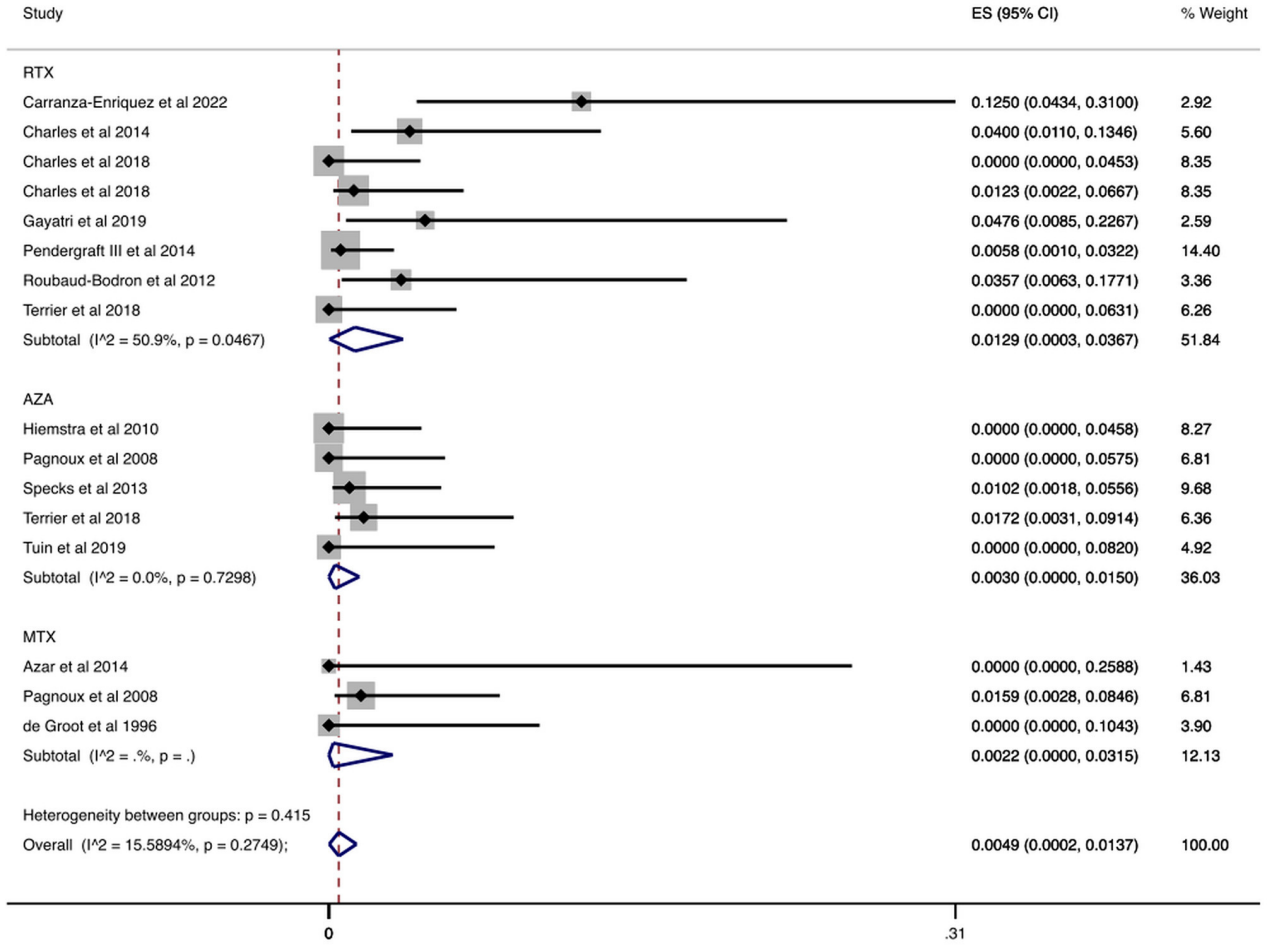
Case fatality rate attributable to infection during the maintenance period. Rate of patients with AAV and serious infection who died from the infection overall and stratified by agent administered during the maintenance period. Individual and combined estimates of the case fatality rate attributable to infection with 95% confidence intervals. ES, Effect Size (Case fatality rate).

We found 963 patients in studies that reported at least one patient with serious infection and recorded any infection-related deaths (7, 30, 32, 33, 36, 38, 40–43, 45, 48–50). We summarize the report on case fatality rates in Figure 4 and Table 2. More specifically, the overall case fatality rate was 0.49% (CI 95%: 0.02–1.37%). Case fatality rate was 1.29% (CI 95%: 0.03–3.67%) in patients treated with rituximab and 0.30% (CI 95%: 0.00–1.50%) in patients treated with azathioprine. In terms of other agents, the case fatality rate of patients treated with methotrexate was 0.22% (CI 95%: 0.00–3.15%).

Case fatality rates remained low when we exclusively examined RCTs (7, 30, 32, 33, 49, 50). Specifically, as we show in Table 2 and Supplementary Figure 1, the overall case fatality rate was 0.27% (CI 95%: 0–1.12%) for 561 patients with relevant data. Additionally, the case fatality rate in patients treated with rituximab was 0.22% (CI 95%: 0.00–1.70%).

#### 3.3.2. Infection-related mortality during the maintenance period

**Figure 5:**
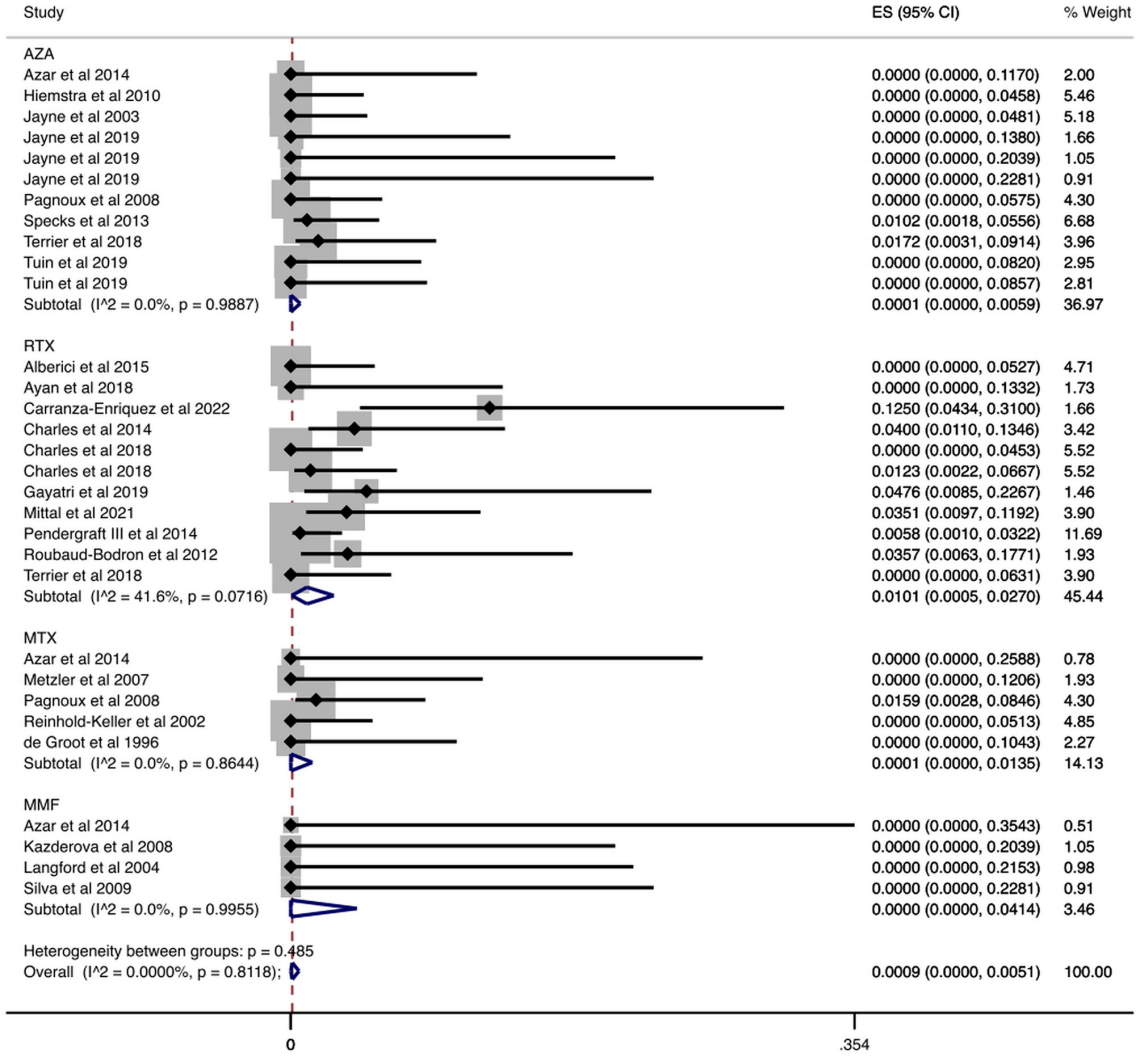
Infection-related mortality rate during the maintenance period. Rate of patients with AAV who died from infection in relation to the total available number of individuals with AAV during the maintenance period. Individual and combined estimates of the infection-related mortality rate with 95% confidence intervals. ES, Effect Size (Mortality rate).

We analyzed data from 1,460 patients who had participated in studies with accessible information on the cause of death over the maintenance period (12–42 months) (7, 30, 32–43, 45, 46, 48–51, 58, 62, 63). In Figure 5 and Table 2, we depict the infection-related mortality rates during the maintenance period. The overall rate was 0.09% (CI 95%: 0.00–0.51%) and when we stratified patients with AAV by agent prescribed, we found low infection-related mortality rates. Mortality rate ranged from 0.01% (azathioprine) to 1.01% (rituximab). With regards to agents used in non-severe disease, mortality rate ranged from 0.00% (mycophenolate mofetil) to 0.01% (methotrexate).

In Table 2 and Supplementary Figure 2 we present that infection-related mortality rates in a separate analysis of data from RCTs (7, 30, 32, 33, 49, 50). More specifically, we identified 821 patients from RCTs who participated in studies which reported the cause of death during the maintenance period (7, 30, 32, 33, 49, 50). The overall infection-related mortality rate was 0.07% (CI 95%: 0.00–0.62%). Stratified by agent, infection-related mortality rate was 0.01% (CI 95%: 0.00–0.63%) with azathioprine and 0.22% (CI 95%: 0.00–1.70%) with rituximab. As for other agents, among patients treated with methotrexate, the infection-related mortality rate was 0.75% (CI 95%: 0.00–4.37%) during the maintenance period.

Among 14 documented infection-related deaths in 23 studies (7, 30, 32–43, 45, 46, 48–51, 58, 62, 63), seven deaths were attributed to respiratory infections. Two out of the seven deaths were due to COVID-19 pneumonia. Finally, sepsis was identified as the cause of death in six patients. One infection-related cause of death remained unspecified (Table 3).

#### 3.3.3. Serious infections during total follow-up

**Figure 6:**
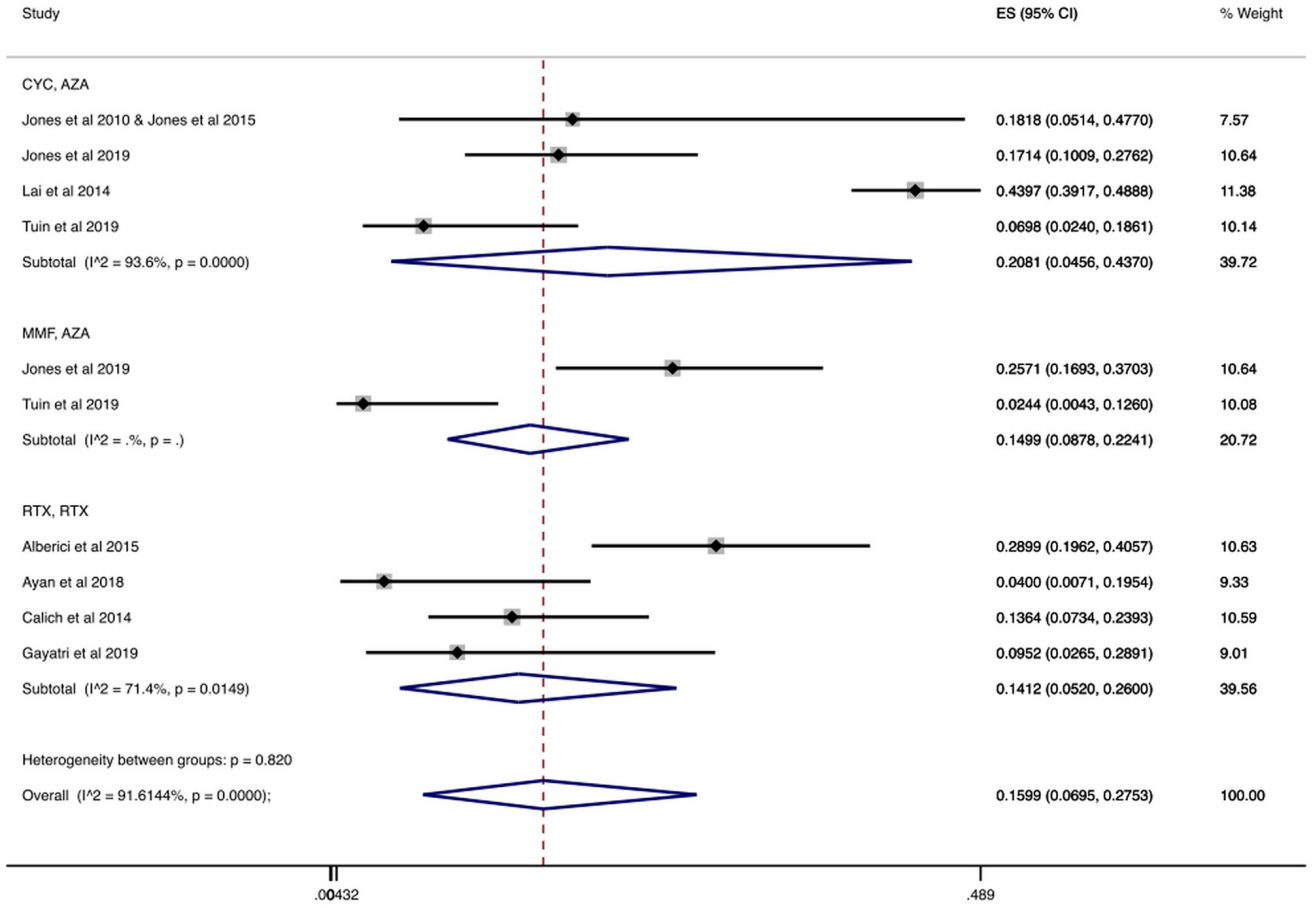
Serious infections cumulative incidence during the total follow-up period. Serious infections overall and stratified by agent administered during the total follow-up period. Individual and combined estimates of the cumulative incidence of serious infections with 95% confidence intervals. ES, Effect Size (Cumulative incidence).

We found 814 patients from studies that reported the number of patients who had serious infections during the follow-up period, which ranged from 18 to 59.3 months (32, 40, 46, 53, 56, 61, 62, 64). In Table 2 and Figure 6 we present the cumulative incidences calculated during total follow-up. In particular, the overall cumulative incidence of serious infections was 15.99% (CI 95%: 6.95–27.53%). Additionally, when we stratified patients with AAV by treatment regimen, the combination of cyclophosphamide for induction and azathioprine for maintenance showed a 20.81% (CI 95%: 4.56–43.70%) incidence of serious infections. Among patients who received rituximab for both induction and maintenance the cumulative incidence of serious infections was 14.12% (CI 95%: 5.20–26.00%).

Also, a sub-analysis was performed regarding the cumulative incidence of serious infections during the total follow-up period only in RCTs (32, 53, 56). As shown in Table 2 and Supplementary Figure 3, when we analyzed 235 individuals enrolled in RCTs, the overall incidence of serious infections during total follow-up was 12.59% (CI 95%: 4.66–23.16%). The cumulative incidence ranged from 12.50% (CI 95%: 5.64–21.19%) in patients treated with cyclophosphamide/azathioprine to 14.99% (CI 95%: 8.78–22.41%) in patients treated with mycophenolate mofetil/azathioprine during total follow-up.

### 3.4. Heterogeneity and quality of individual studies

We utilized the RoB 2 and ROBINS-I tools to assess RCTs and observational studies for quality, respectively. We deemed all RCTs to have low risk of bias across the domains of randomization process, deviation from the intended interventions, extent of missing outcome data, outcome measurement, outcome selection, as well as overall. Additionally, we found that 4 observational studies had a low, 7 had a moderate, and 3 had a high risk of bias. We present the detailed quality assessment data in the Supplementary Figures 4, 5. Regarding heterogeneity, the I2 values of our outcomes overall ranged from low to high (0.00–91.61%). Egger's test for publication bias showed no evidence of small-study effects (coefficient:−0.457, *p*-value: 0.190). Overall, the certainty of evidence in our findings ranged from very low to high. The quality of evidence for each of our outcomes and their respective stratifications is summarized in the Supplementary Table 2.

## 4. Discussion

Severe infections are a major complication in patients with AAV (3). In this meta-analysis we included data from 2,938 patients who were followed for 12 to 64.8 months. We found that the cumulative incidence of serious infections was 7.62% during the maintenance period, and 15.99% during the total follow-up period. Importantly, we found a numerically higher cumulative incidence of serious infections among patients treated with cyclophosphamide and azathioprine than patients treated with rituximab for both induction and maintenance, while the case fatality and infection-related mortality rates were low, overall and across different maintenance regimens. Documented serious infections primarily involved the respiratory track, while infection-related mortality is usually associated with sepsis or pneumonia.

According to the treatment guidelines by the American College of Rheumatology, in patients with severe AAV rituximab is the preferred agent for both induction (over cyclophosphamide) and maintenance (over methotrexate, azathioprine, or mycophenolate mofetil) of remission (4). Primarily for induction of remission with rituximab, a recent meta-analysis of 1,434 patients with AAV treated with rituximab found a 15.40% incidence of serious infections and as follow-up lengthened, the incidence appeared to decrease (14). Additionally, while we could not directly compare two different treatment regimens, the cumulative incidence of serious infection in patients receiving rituximab was numerically lower than the incidence of serious infections in patients receiving cyclophosphamide/azathioprine for the total follow-up period (induction and maintenance). Our analysis indicates that patients receiving a rituximab treatment scheme for both induction and maintenance did not experience an increase in serious infections throughout the treatment period compared to the reported incidences during induction of remission.

Overall, we found an infection-related mortality during the maintenance period lower than 1%. Our reported infection-related mortality rates confirmed that, regardless of the agent used, fatal infections are uncommon during maintenance. This has also been evident in a long-term follow-up study with 476 participants, where after the first year of treatment, 15 (15/476, 0.03%) of reported deaths were caused by infection (3). In the past, reported infection-related mortality rates during the maintenance period were more than 1.5% (65). Evidently, we showed that fatal infections are decreasing as newer immunosuppressive agents for maintenance are introduced and cyclophosphamide is used less frequently. However, it remains to be seen, whether the very low rates of infection-related mortality are also attributed to experience in the management of infectious complications in this patient population.

Respiratory infections, especially pneumonia, comprised half of serious infection causes reported. We found pneumonia and sepsis as the most significant causes of fatal serious infection in patients with AAV. Similarly, the findings of a previous long-term follow-up study attributed 15/15 infection-related deaths to either pneumonia or sepsis after 1 year of treatment (3). Therefore, increased vaccination compliance and additional prophylactic measures are required during the COVID-19 pandemic given the increased risk of death due to respiratory infections in patients living with rheumatologic diseases (66).

Notably, two cases of *Pneumocystis jirovecii* pneumonia were identified in RCT participants who had been thoroughly screened and/or were receiving antibiotic prophylaxis. Therefore, increased vigilance for *Pneumocystis jirovecii* pneumonia is required in patients with AAV. Recent European League against Rheumatism guidelines emphasize the importance of antibiotic prophylaxis against *Pneumocystis jirovecii* infection in patients receiving high glucocorticoid doses, particularly in patients with AAV (67). Further studies are needed to determine the role of some patient characteristics and immunosuppressive regimens as risk factors.

Regarding limitations of our study, we included both RCTs and observational studies. To address this limitation, we performed a sub-analysis that was based only on data from RCTs and confirmed that our findings did not change. In addition, we considered cohorts in which rituximab treatment was concomitantly prescribed with other immunosuppressive treatment to be under our rituximab sub-group. Of note, for patients treated with RTX during the maintenance period, we were unable to perform a meta-regression analysis for their cumulative RTX dose or the proportion of patients who developed hypogammaglobulinemia due to a lack of sufficient available data in the included studies. Also, we were unable to calculate any correlation of glucocorticoid dosage with serious infections incidence because there was not a standardized way of reporting the dosage during maintenance. Furthermore, any correlation of total follow-up or maintenance duration with the incidence of serious infections could not be examined. This was because there was no individualized method of reporting the duration of total follow-up and maintenance treatment, which could lead to ecological fallacy (68). Finally, some cohorts were entirely composed of patients with GPA. To combat this concern, we performed meta-regression analysis which indicated that the difference in the proportion of patients with GPA or MPA did not significantly affect our findings.

## 5. Conclusions

Although rituximab is now recommended for both induction and maintenance of remission, serious infections remain among the most common complications in patients with AAV. We found that rituximab does not increase the incidence of serious infections, while also being effective especially in patients with severe disease. Furthermore, our findings substantiated the fact that available maintenance treatment agents have very low case fatality and infection-related mortality rates. In order to corroborate these findings, clinical practice with close monitoring of serious infections during prolonged follow-up are needed.

## Data availability statement

The original contributions presented in the study are included in the article/Supplementary material, further inquiries can be directed to the corresponding author.

## Author contributions

AV, SV, MK, FS, and EM conceptualized and designed the study and participated in data interpretation. AV and SV participated in data collection and extraction. AV and FS prepared tables and figures and performed the statistical analysis. AV drafted the initial manuscript. SV, MK, FS, and EM reviewed and revised the manuscript. All authors have read and approved the final manuscript as submitted and agreed to be accountable for all aspects of the work in ensuring that questions related to the accuracy or integrity of any part of the work are appropriately investigated and resolved.
