# Supplementary material for: Incidence of serious infections in patients with ANCA-associated vasculitis receiving immunosuppressive therapy: A systematic review and meta-analysis
Source: Front Med (Lausanne). 2023 Mar 1;10:1110548. doi: 10.3389/fmed.2023.1110548 (PMC10014619; doi:10.3389/fmed.2023.1110548)

Supplementary Appendix

Supplement to: Incidence of Serious Infections in Patients with ANCA-associated Vasculitis Receiving Immunosuppressive Therapy: A Systematic Review and Meta-Analysis

Contents

Section 1: Supplemental Figures

Table S1: Meta-regression analysis

Table S2: GRADE quality of evidence of primary and secondary outcomes

Figure S1: Case fatality rate attributable to infection during maintenance period in RCTs

Figure S2: Infection-related mortality rate during the maintenance period in RCTs

Figure S3: Cumulative incidence of serious infections during total follow-up in RCTs

Figure S4: Individual study risk of bias in each domain and overall, for RCTs

Figure S5: Individual study risk of bias in each domain and overall, for observational studies

Table S1 Meta-regression analysis

Variables	Coefficient	p-value	95% CI
GPA	-0.030	0.311	-0.089 - 0.030
MPA	-0.026	0.392	-0.088 - 0.036
Patient Number	0.029	0.329	-0.160 - 0.312

Table S2 GRADE quality of evidence of primary and secondary outcomes

Outcome	Number of patients	Treatment arms in RCTs/Observational Studies	Risk of Bias *	Inconsistency (I^2) **	Indirectness	Imprecision	Publication bias	Effect size	Dose Effect	GRADE
**Incidence of serious infections during the maintenance period**										
Overall	1,284	11/27	High↓↓	76.98%↓	Not detected	Not detected	Not detected	NA	NA	**Very low**
AZA	412	6/7	Low	83.30%↓	Not detected	Not detected	Not detected	NA	NA	**Moderate**
RTX	617	3/11	High↓↓	56.50%	Not detected	Not detected	Not detected	NA	NA	**Low**
MTX	206	2/5	Low	68.10%	Not detected	Not detected	Not detected	NA	NA	**High**
MMF	49	0/4	High↓↓	0%	Not detected	Not detected	Not detected	NA	NA	**Low**
Overall RCTs	693	11	Low	81.93%↓	Not detected	Not detected	Not detected	NA	NA	**Moderate**
AZA RCTs	383	6/0	Low	83.90%↓	Not detected	Not detected	Not detected	NA	NA	**Moderate**
RTX RCTs	219	3/0	Low	NA↓	Not detected	Not detected	Not detected	NA	NA	**Moderate**
MTX RCTs	91	2/0	Low	NA↓	Not detected	Not detected	Not detected	NA	NA	**Moderate**
**Case fatality rate during the maintenance period**										
Overall	963	9/16	High↓↓	15.59%	Not detected	Not detected	Not detected	NA	NA	**Low**
RTX	514	3/8	High↓↓	50.90%	Not detected	Not detected	Not detected	NA	NA	**Low**
AZA	342	5/5	Low	0%	Not detected	Not detected	Not detected	NA	NA	**High**
MTX	107	1/3	High↓↓	NA↓	Not detected	Not detected	Not detected	NA	NA	**Very low**
Overall RCTs	561	8/0	Low	0%	Not detected	Not detected	Not detected	NA	NA	**High**
RTX RCTs	219	3/0	Low	NA↓	Not detected	Not detected	Not detected	NA	NA	**Moderate**
AZA RCTs	342	5/0	Low	0%	Not detected	Not detected	Not detected	NA	NA	**High**
**Infection-related mortality rate during the maintenance period**										
Overall	1,460	15/31	High↓↓	0%	Not detected	Not detected	Not detected	NA	NA	**Low**
AZA	540	10/11	Moderate↓	0%	Not detected	Not detected	Not detected	NA	NA	**Moderate**
RTX	665	3/11	High	41.60%	Not detected	Not detected	Not detected	NA	NA	**Low**
MTX	206	2/5	Low	0%	Not detected	Not detected	Not detected	NA	NA	**High**

Outcome	Number of patients	Treatment arms in RCTs/Observational Studies	Risk of Bias *	Inconsistency (I^2) **	Indirectness	Imprecision	Publication bias	Effect size	Dose Effect	GRADE
MMF	49	0/4	High↓↓	0%	Not detected	Not detected	Not detected	NA	NA	**Low**
Overall RCTs	821	15/0	Low	0%	Not detected	Not detected	Not detected	NA	NA	**High**
AZA RCTs	511	10/0	Low	0%	Not detected	Not detected	Not detected	NA	NA	**High**
RTX RCTs	219	3/0	Low	NA↓	Not detected	Not detected	Not detected	NA	NA	**Moderate**
MTX RCTs	91	2/0	Low	NA↓	Not detected	Not detected	Not detected	NA	NA	**Moderate**
**Incidence of serious infections total follow-up period**										
Overall	814	5/10	Low	91.61%↓	Not detected	Not detected	Not detected	NA	NA	**Moderate**
CYC, AZA	522	3/4	Low	93.60%↓	Not detected	Not detected	Not detected	NA	NA	**Moderate**
MMF, AZA	111	2/2	Low	NA↓	Not detected	Not detected	Not detected	NA	NA	**Moderate**
RTX, RTX	181	0/4	Low	71.40%↓	Not detected	Not detected	Not detected	NA	NA	**Moderate**
Overall RCTs	235	5/0	Low	74.20%↓	Not detected	Not detected	Not detected	NA	NA	**Moderate**
CYC, AZA RCTs	124	3/0	Low	NA↓	Not detected	Not detected	Not detected	NA	NA	**Moderate**
MMF, AZA RCTs	111	2/0	Low	NA↓	Not detected	Not detected	Not detected	NA	NA	**Moderate**

*: If moderate or high risk of bias was present in at least one included study, quality of evidence was downgraded by 1 or 2 levels, respectively.
**: Quality of evidence was downgraded by one level if I^2 was higher than 70% or not available.
↓: Reduction of “quality of evidence” by 1 level.
↓↓: Reduction of “quality of evidence” by 2 levels.
AZA: azathioprine, CYC: cyclophosphamide, GRADE: Grading of Recommendations, Assessment, Development, and Evaluation
MMF: mycophenolate mofetil, MTX: methotrexate, NA: not available RCT: randomized clinical trial, RTX: rituximab

Figure S1 Case fatality rate attributable to infection during maintenance period in RCTs

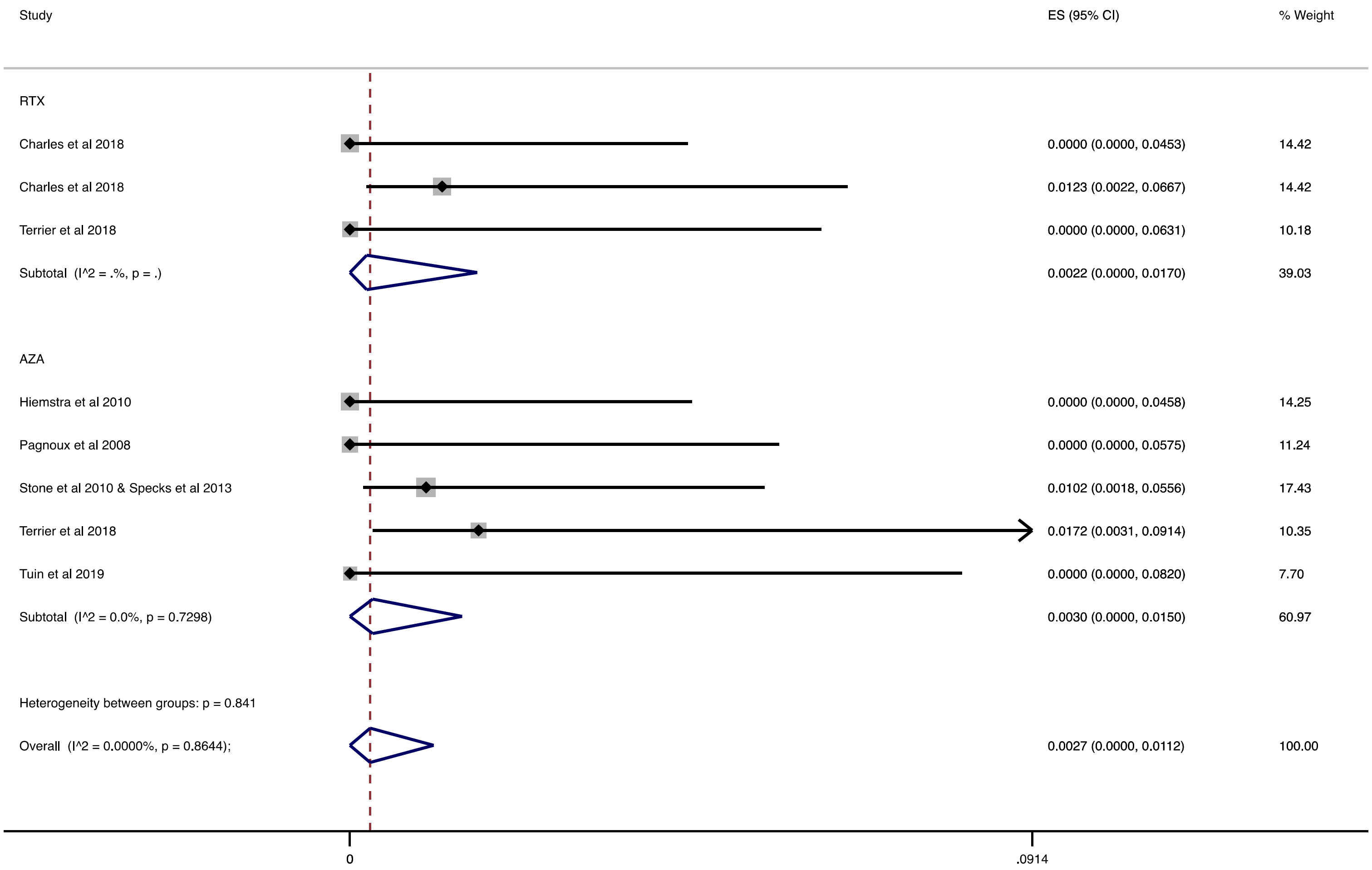

Figure S2 Infection-related mortality rate during the maintenance period in RCTs

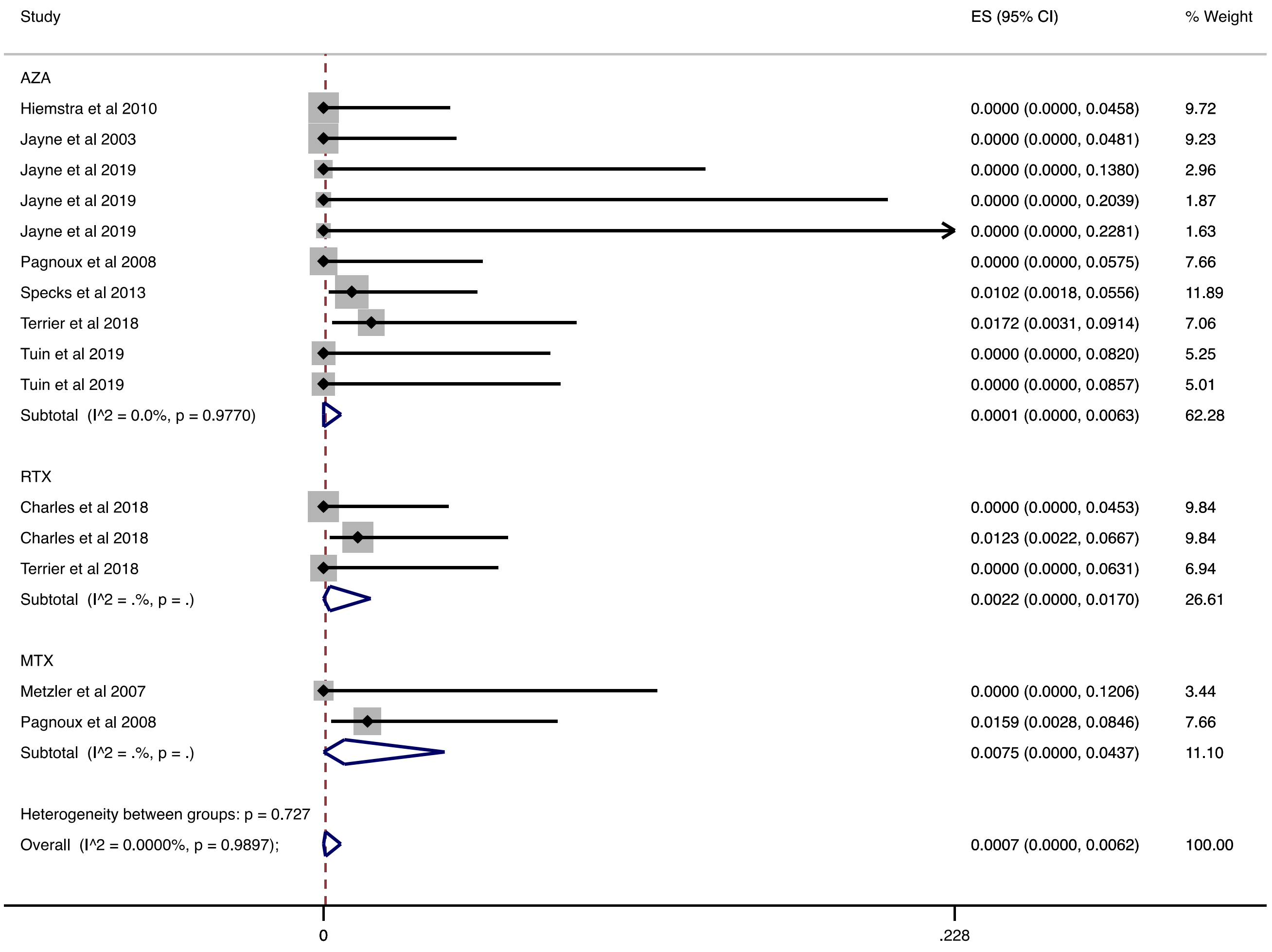

Figure S3 Cumulative incidence of serious infections during total follow-up in RCTs

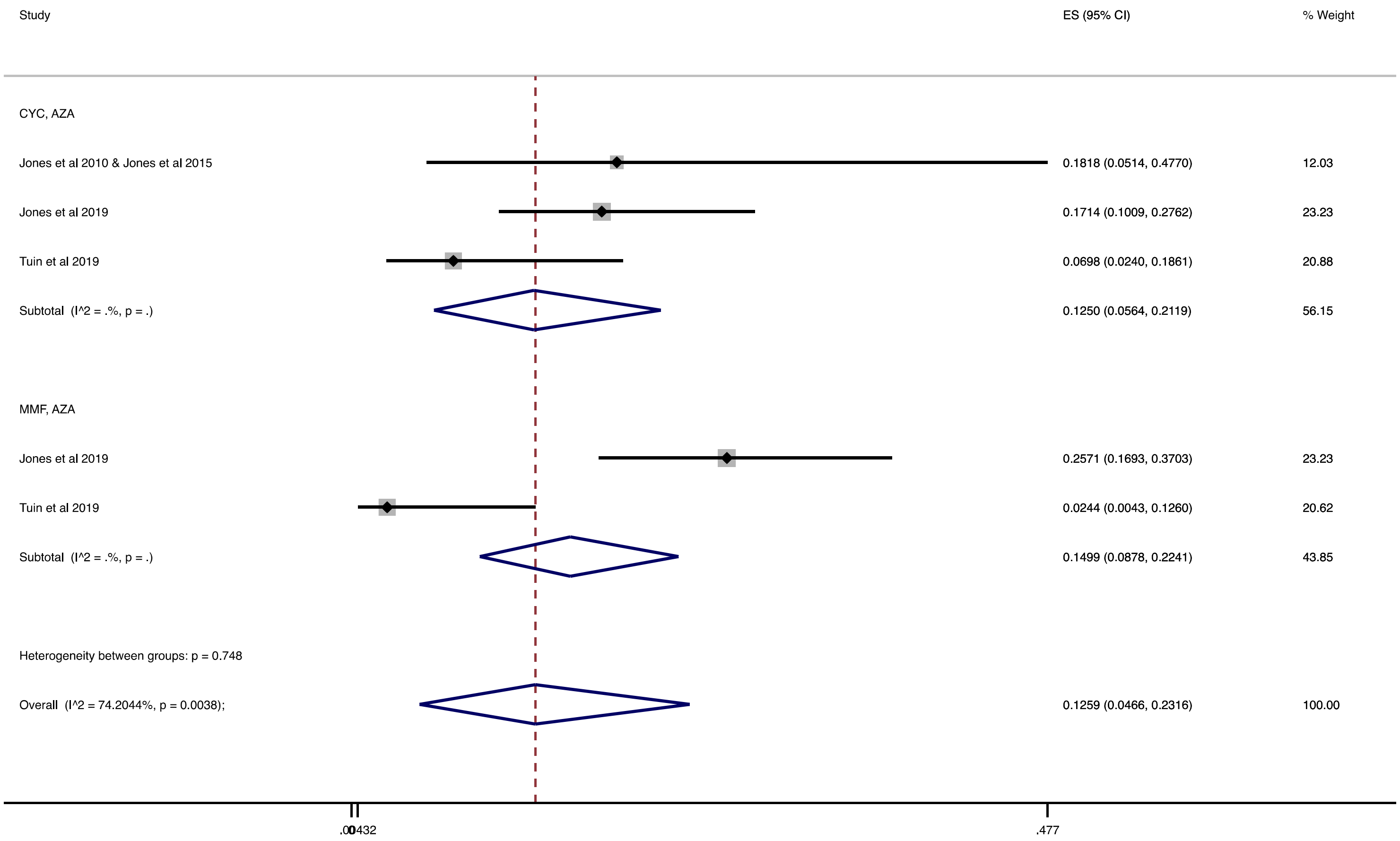

Figure S4 Individual study risk of bias in each domain and overall

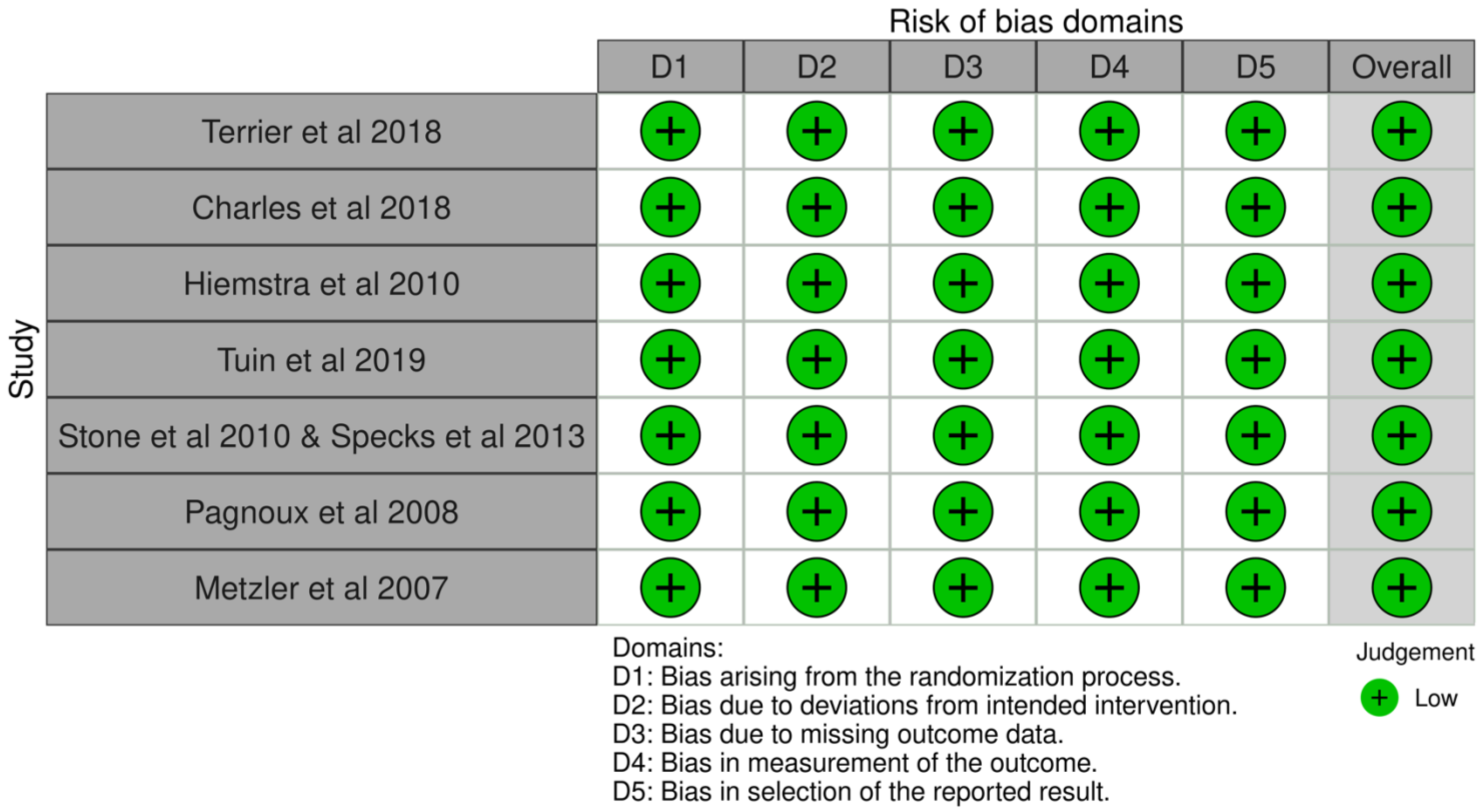

Figure S5 Individual study risk of bias in each domain and overall, for observational studies

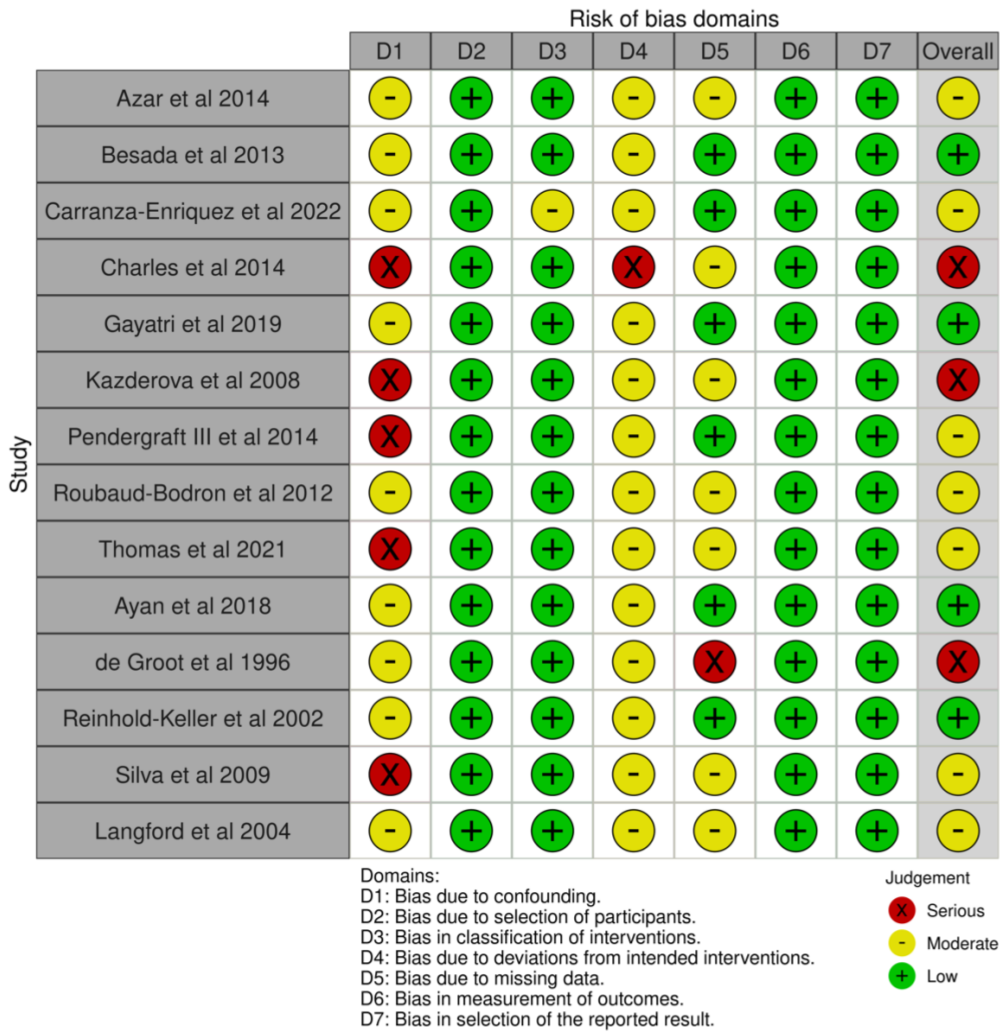

Supplement: Supplementary file 1 [file Data_Sheet_1.PDF]
